# Supplementary material for: Access to domestic violence advocacy by race, ethnicity and gender: The impact of a digital warm handoff from the emergency department
Source: PLoS One. 2022 Mar 18;17(3):e0264814. doi: 10.1371/journal.pone.0264814 (PMC8932576; doi:10.1371/journal.pone.0264814)
Supplement: S1 Appendix — (PDF) [file pone.0264814.s001.pdf]

Appendix: Standardized Data Abstraction Form

Use these guidelines to enter data into the corresponding Excel sheet cells for each patient

Visit ID

This is the number associated with the patient's visit. A single patient may have multiple Visit IDs if they visited the hospital multiple times We will be taking out repeat patients..

Date of patient visit

The date of the patient visit is collected as indicated in the patient's chart, then, for the final data set, is rounded to the first day of that week to protect patient confidentiality.

Patient age

Indicated in years, as listed in patient chart on date of visit.

Patient race / ethnicity

Z = Other Race (often chosen for Hispanic)
O = Other Asian
B = Black
W = White
C = Chinese
A = American Indian/Alaska Native
F = Filipino
I = Asian Indian
U = Unknown/Declined to State
V = Vietnamese
H = Native Hawaiian
S = Samoan
P = Other Pacific Islander
J= Japanese
K= Korean
G= Guamanian/Chamarro

Patient sex/gender

Indicated as written in patient chart.

F=Female

M=Male
X=Other

Perpetrator

If patient chart indicated violence, assault, or abuse, we recorded the perpetrator as written in the chart or from their mandatory report form.

If multiple perpetrators are listed, include all that are IPV/DV relationship.

1. If different perpetrators are listed by different providers write the relationship with the greatest consensus, or the one that indicates the greatest degree of disclosure (e.g., fiance > boyfriend, ex-husband > husband).
2. Multiple perpetrators label with “and” (“husband and brother-in-law”); same perpetrator with multiple roles label with “/” (“roommate / father of baby”).
3. In the situation where there is conflicting information with no greater degree of disclosure (ex: husband vs. boyfriend) note down what the social worker added to pt’s file

Boyfriend
Husband
Girlfriend
Wife
Partner
Ex-Boyfriend
Ex-Girlfriend
Coworker
Stranger(s) = Patient does not know the person who assaulted them
Mother
Father
Uncle
Aunt
Roommate
Nephew
Niece
Grandson
Granddaughter
Acquaintance

NA = Not applicable; the case didn’t have a perpetrator (i.e. wasn’t an assault)
Unknown = The file doesn’t say who assaulted them OR unsure of relationship to patient

**Sexual Assault?**<br><br>Whether or not sexual assault was indicated or recorded in patient chart.<br><br>Y=Sexual assault indicated<br>N=No (pt in explaining the situation explicitly said there was no sexual assault, sexual assault never mentioned in pt chart)
**Screened for DV/safety at home/abuse**<br><br>This indicates whether screening questions were asked by a provider to the patient, results are written in "Triage" section of the patient's chart.<br><br>Y=screening questions and result present in chart<br>N=screening questions and result missing in chart
**Screening result (DV or No DV)**<br><br>The screening results of patients, as indicated in the "Triage" section of the patient's chart.<br>1=Are you being physically hurt or threatened by someone close to you in your living situation?<br>2=Has Social Services been notified?
**DV/IPV was specified in triage notes as chief complaint?**<br><br>This indicates that the patient's chief complaint was either Domestic Violence or Intimate Partner Violence. This is indicated by the patient's chart reporting "Chief Complaint" as "assault" or "domestic violence". This is also implied if during triage the patient explicitly tells the nurse that their injuries are a result of DV or IPV.<br><br>N = The patient's chief complaint is not DV/IPV<br>DV = The patient's chief complaint is a result of Domestic Violence<br>IPV = The patient's chief complaint is a result of Intimate Partner Violence
**Notes indicated DV or IPV**<br><br>Came in for unrelated/non DV/IPV reason, but turned out to experience DV/IPV). The notes in the patient's file stated the occurrence of DV/IPV, indicated through nurse's, doctor's, or social worker's notes, regardless of whether they screened positive or negative during the "Abuse Screen".

N = Notes didn't indicate DV/IPV<br>DV= Notes indicated DV<br>IPV = Notes indicated IPV
**Given referral to DV/IPV services?**<br><br>The patient chart/forms filed noted that referrals were given (may or may not have specified in notes).<br><br>Y= Yes, resources were given (specified in chart or mandatory report)<br>N= No, resources were not given; no indication or notes regarding any resources
**Nature of referral(s) given**<br><br>This indicates the type of referrals and resources the patient was provided with.<br><br>PD = Police Report Filed -- If PD notified written in Triage, just assume hospital called PD not PTA<br>J= Pt in police custody (possibly being medical cleared for jail). No further resources provided.<br>OTHER = Other resources provided (homeless counselor, health advocates, detox center, motel voucher, etc)<br><br>**Psych Services:**<br>5150 = Placed on Psychiatric Hold (Danger to self/others)<br>JGP = Pt transferred to psychiatric hospital<br>MHR = Other mental health resources<br><br>**Legal Services:**<br>EPO = Emergency Protective Order<br>STO = Stay Away Order<br>RO = Restraining Order, restraining order info/advice<br>VOC = Victim of Crime<br>OLA = Other Legal Aid<br><br>**Social Services:**<br>SS= Talked with a social worker, social worker provided resources/referrals<br>APS = Adult Protective Services<br>CPS = Child Protective Services

FJC = Family Justice Center
FVLC = [Region-specific DV resource]
FIS = [Region-specific DV resource]
ODV= Other Unspecified DV Resources (hotlines, community resources, counseling, DV Risk Assessment, etc.)

SH = Shelter Resources
ED = Education for DV/IPV
TRAN = Transportation (bus pass, taxi voucher, money given for transportation)
PP = Planned Parenthood ed

Sexual Assault/Rape Services:
SART = Sexual Assault Response and Resources Team (261 exam) **note all SART exams include an advocate**
BAWAR = [Region-specific sexual assault resource]
THV = [Region-specific sexual assault resource]
OSA = Other Sexual Assault Services/ Referrals
AD = Advocate provided additional resources (more than just being present during 261 exam)

Services Refused/Unable to be Provided:
DNP = Decline police interaction (any time -- decline to call, decline to speak with, decline to give information)
DNDV = Declined DV Resources/Services (NOTE: DNDV is treated as another referral category and can be added onto other resources. DNDV is more common at HGH than SR. DNDV only appears in post-DVRR records at SR)
DSART = Declined SART services
DSS = Declined social worker services

EL: Patient Eloped (includes AMA)
NTS: No timely service (services took too long/not available for follow up, (social worker was contacted, paged, or notified, but no indication that they came or followed up)
N/A = No referrals or resources were provided

MPF System

This indicates whether or not a patient has a confidential (mandatory) report in their MPF file. This is indicated in the "Forms Completed" section of the abuse screen.

Note: Only put yes if the forms completed in MPF are DV related. Some reports are 5150 or consent related, and some confidential reports are rabies related. Note that 2018 patients may not have MPF forms filed in time for us to know about them (by 4/2/18)<br><br>Y = the patient has a confidential report form in the MPF system<br>N = the patient does not have a confidential report form in the MPF system
**Given additional resources (MPF)?**<br><br>This indicates whether or not additional DV resources were provided to the patient. On the confidential report form this is indicated as a check box for the question, “Were additional DV resources provided?” On the new DVRR form this is indicated as explicit written text input by the healthcare provider filling out the form.<br><br>Y = Confidential report: Additional DV resources were provided to the patient as indicated on the checkbox on their confidential report in their MPF file.<br>DVRR form: If there is no confidential report but there is a DVRR form (on which there is no explicit check box for additional resources), put yes if the file explicitly mentions giving the patient additional resources.<br><br>N = Additional DV resources were NOT provided to the patient as indicated on their confidential report in their MPF file. If the file on PICIS states that additional resources were given, but no additional resources were given on MPF, put no. (I.e. looking through the file for additional resources only applies to DVRR forms)<br><br>NA = Patient does not have forms in the MPF system OR patient only has DVRR form and there is no explicit mention of giving additional resources.
DVRR system<br><br>This variable indicated whether or not a patient had a DVRR electronic form filled out for them by a healthcare provider in the ED.<br><br>Y = The patient had a DVRR form filled out by ED staff and they have a file in the DVRR system<br>N = The patient did not have a DVRR form filled out by ED staff and they are not in the DVRR system
**Mandatory report sent**

Indicates whether a mandatory police report was filed on behalf of the patient.

Y = Yes (DV/IPV reports)
N = No (no DV/IPV report filed when indicated)
PAC = DV/IPV case; police already contacted prior to the patient coming to the ED. Must be indicated in notes.
NA = non-applicable (for perpetrators -- everyone that has a J becomes NA in Mandatory Report)
OR=Other report. These fall under OR:

- 5150 = Psychiatric mandatory reports (add PTA if prior to arrival)
- 261 = SART mandatory reports
- APS = Adult Protective Services
- CPS = Child Protective Services
- NR = No report needed for this patient, not relevant case, came in for reasons unrelated to DV
