# Supplementary material for: Access to domestic violence advocacy by race, ethnicity and gender: The impact of a digital warm handoff from the emergency department
Source: PLoS One. 2022 Mar 18;17(3):e0264814. doi: 10.1371/journal.pone.0264814 (PMC8932576; doi:10.1371/journal.pone.0264814)
Supplement: S2 Appendix — Domestic Violence Report and Referral: A Multiple Baseline Study of an eHealth Warm Handoff for Emergency Department Patients affected by Domestic Violence. (PDF) [file pone.0264814.s002.pdf]

Domestic Violence Report and Referral: A Multiple Baseline Study of an eHealth Warm Handoff for Emergency Department Patients affected by Domestic Violence

Abstract (248 words)

Objective: Emergency department (ED) interventions for domestic violence (DV) rarely connect patients to community-based advocacy services. This causal analysis explores changes in advocacy receipt after implementation of a novel eHealth intervention.

Methods: In this multiple baseline study, we reviewed 2292 DV-related patient visits at three Northern California EDs and 461 subsequent visits to a large DV advocacy agency between 2013-2018. Between 2014-2016, each ED implemented a digital direct-to-advocacy referral system, Domestic Violence Report and Referral (DVRR), which providers used at their discretion. We used generalized linear models stratified by ED to identify significant changes in patient likelihood of receiving advocacy services associated with DVRR. We tested for external and internal validity using Maentel-Haenszel weighted chi-square tests, internal validity using one-way MANOVA, and external validity using visual multiple baseline analysis and logistic regression.

Results: DVRR was associated with significant increases in advocacy services. When the intervention was implemented, the relative proportion of patients reaching DV advocacy increased by 1.34 (95% CI: 1.05-1.71) to 3.55 (95% CI: 1.61-7.91) times in the three study EDs. Survivors who received the intervention reached advocacy at 2.55 (95% CI: 2.13-3.05) to 6.16 (95% CI: 2.93-12.95) times the proportion of non-intervention patients. External validity tests suggest that changes were localized to the time and place of the intervention; internal validity tests suggest that the intervention impacted DV referrals significantly more than non-DV referrals.

Conclusion: These findings suggest direct-to-advocacy referrals improve rates of DV-affected patients' connection to advocacy.

Introduction

Background

Intimate partners inflict violence on approximately 6.6 million U.S. women each year.[1] For these women, hospital emergency departments (EDs) are often a key point of receiving care. Each year from 2006-2009, EDs across the U.S. coded approximately 28,000 visits for domestic violence (DV), which may include violence perpetrated by intimate partners or other family or household members.[2] This is likely to be an underestimate. While national data is sparse, individual hospitals' recent data estimates that DV affects between 3-21% of female ED patients.[3–7] In addition, among these survivors, a recent point estimate suggests that 61% of these survivors were at high or extremely high risk of intimate partner homicide.[8,9]

EDs represent a crucial point of interaction for these patients. Yet a systematic review of guidance around DV interventions in EDs are ambiguous and inconsistent, and typical non-medical DV interventions in EDs, such as universal screening, are inefficient at reducing future DV victimization.[10] A systematic review of 30 studies noted that DV screening neither improves patient quality of life nor decreases future DV victimization when compared to the acute treatment of injuries alone.[11] Some EDs also provide written educational material or lists of relevant service agencies to patients affected by DV. A randomized controlled trial of 2708 DV patients found that these materials had no impact on future hospitalization, ED visits, or DV advocacy agency contact at one- or three-year follow up.[12,13] As of 2019, 44 states mandate medical providers to report at least some DV to law enforcement, and 13 states mandate medical providers to report all injuries caused by DV.[14] Yet a systematic review found only sparse, dated empirical research on the effectiveness of mandatory reporting in reducing DV, and the included studies reported mixed and often biased findings.[15]

Importance of "Warm Handoffs" and eHealth Tools

Specialist counseling, structured risk assessment, safety planning, and other evidence-based DV interventions may mitigate the risks of harm to DV-affected ED patients.[16] Specialist counseling may include direct personal contact within the ED or via ED staff to a named DV advocate, a practice known as a "warm handoff." The Agency for Healthcare Research and Quality considers warm handoffs a best practice for coordinating care between healthcare and social services.[17] Several randomized controlled trials highlight the efficacy of warm handoffs in connecting patients to care and improving relevant outcomes in non-DV settings.[18–20] While fairly novel in ED settings, individual EDs that have implemented a warm handoff approach to connecting patients to DV advocacy report improved patient connections to needed non-medical care.[6,17,21]

Electronic, or eHealth, tools at individual hospitals typically convert existing interventions, such as screening protocols, education materials or referral lists, into digital formats.[22–26] A systematic review of 31 such interventions found that eHealth interventions streamlined traditional interventions but did not improve upon their outcomes.[27] eHealth interventions may facilitate promising interventions such as warm handoffs to advocacy. A cluster-randomized trial in the United Kingdom found that when primary care providers sent digital referrals directly to professional DV advocates, the odds of patients receiving DV referral services increased threefold.[23]

Goals of this Investigation

This multiple baseline study examines the effect of a digital warm handoff intervention, the Domestic Violence Report and Referral (DVRR), on DV referrals and advocacy services for patients in three Northern California EDs. DVRR, described more fully below, sends digital referrals to advocates on behalf of consenting patients, who then reach out to the referred patient. DVRR combines this novel referral protocol with simultaneous digital DV reports to law enforcement as mandated by the state of California. This study focuses on the advocacy referral pathway and the impact of the intervention on DV referrals and advocacy services.

Methods

Study Design and Setting

This study uses a mixed non-concurrent and concurrent multiple baseline design. Multiple baseline designs are an alternative to randomized controlled trials for situations where withholding an intervention from a control group may not be feasible or ethical, or where researchers want to consider individuals or groups as their own controls.[28,29] Within this design, researchers make repeated measurements of multiple individuals or groups while the intervention is introduced at different time points for each group. Causal inference is supported when intervention effects follow the intervention starting point within each group and are not simultaneously observed in other groups.[28,30,31] Within public health research, this method is primarily feasible in samples where large effect sizes are anticipated or observed.[28]

Data were collected from two sources: chart reviews of eligible patient visits to one of three participating Northern California EDs (referred to as index visits), and client records at a DV advocacy agency within six months of the index visit. If a patient visited the ED and met inclusion criteria for the study multiple times, that patient may have had multiple index visits and advocacy visits included in the data. The three participating EDs implemented the intervention in February 2014 (Hospital 1), April 2016 (Hospital 2), and May 2016 (Hospital 3), providing a natural basis for a multiple baseline design.

Due to the continuous nature of patient index visits and the delay between index and advocacy visits, main effects were assessed using a non-concurrent multiple baseline approach, in which data are collected repeatedly over multiple time points.[32] We assessed internal and external validity at baseline and after each intervention time point using a concurrent (therefore more robust) multiple baseline approach.[32] Providers at all three hospitals were given discretion as to whether a patient would receive the intervention or the hospital's prior standard of care. As a result, analyses also assessed whether patients who were given the intervention were more likely to reach advocacy than those who were not.

The Committee for the Protection of Human Subjects at a large public university approved the study protocol; the Institutional Review Boards of Hospitals 1 and 3 also approved the protocol while Hospital 2 accepted the university review as sufficient.

Selection of Participants

Eligible ED visits included patients 18 years or older seen at participating EDs between December 2013 and September 2017 whose charts indicated DV in the diagnosis, chief complain,t or medical/social work notes. We used this subset of all patients with a

positive DV screen for consistency across hospitals because each of the three study hospitals used a different DV screening protocol and each offered a different definition of a positive screen.

A prolonged baseline at Hospital 1 was screened beginning February 2013 and a prolonged tail was screened at Hospitals 1 and 2 through April 2018. Patient charts were screened for eligibility using hospital database settings and search functions, with a limited exception at Hospital 2. Hospital 2's database screening was unavailable for the data range December 2013-April 2016. As a result, the research team created a customized program to screen all ED charts during this range for eligibility. In a test using three months of data (June 2016-September 2016), the program screened over 10,000 ED records to identify eligible records, with 99.8% accuracy compared to hospital database screening.

Figure 1 describes the selection flow for patient visits entering the study sample. There were 1553 eligible ED visits at Hospital 1, 385 at Hospital 2, and 101 at Hospital 3. Advocacy agency staff reviewed all eligible visits to identify any contact with these patients within six months of their index ED visit; this resulted in 421 matched records from the advocacy agency for the range February 2013 through September 2018.

Intervention

DVRR is an eHealth program that connects DV-affected patients to advocacy services and law enforcement. It incorporates the Danger Assessment, a validated tool that indicates a patient's risk of intimate partner homicide,[8] as well as information about the assault, a body map to document the patient's injuries, space for medical notes to describe pertinent information about each injury, and, with patient consent, an option to take pictures of the injuries. The health care provider uses DVRR to send a digital copy of the report to local law enforcement as mandated under California law. With patient consent, the health care provider uses DVRR to also send a condensed report that includes the patient's name, their Danger Assessment score, and a safe phone number to a local advocacy agency. Within two weeks of the ED visit, the advocate calls the patient to offer assistance.

Measures

Once eligible charts were identified, members of the research team reviewed them to collect study data. This included patient name, gender, age, race, ethnicity, the result of DV screening, chief complaint, any indication of DV in medical or social work notes, relationship to the perpetrator, any presence of sexual assault, any prior visits to the ED, and the nature of any referrals that had been administered. DV screening protocols differed by hospital; the screening protocols at Hospital 2 and Hospital 3 also included patients who felt unsafe for other reasons, such as homelessness or sexual assault by an unknown perpetrator. As a result, the study only considered confirmed DV cases to include patients who had a chief complaint or diagnosis of DV or had medical/social work notes that indicated DV. At Hospitals 1 and 3, providers categorically offered patients whose victimization included sexual assault medical and psychosocial treatment through their Sexual Assault Response Team (SART). We measured the proportion of DV cases with SART involvement, as this introduced additional referrals and pathways to advocacy contact for affected patients.

At Hospitals 1 and 2, members of the research team trained in medical record-keeping systems and data collection abstracted chart data into a standardized form. The interrater agreement, tested biweekly, averaged 0.93, with a minimum agreement of 0.7 in any abstraction field in any test. Hospital 3 and the advocacy agency staff abstracted their own records using standardized forms provided by the researchers. We used a second customized macro to collect data for selected standardized fields (gender, race, ethnicity, the result of DV screening) within eligible charts at Hospital 2. In a test of accuracy, this program collected data from 157 medical records with 100% accuracy compared to manual data collection. Both programs were created with Pulover's Macro Creator 5.0.5. All other data were collected through manual chart reviews.

After data were abstracted from all three hospitals, the researchers provided the advocacy agency staff the visit dates and first and last names associated with all eligible patient visits. The agency staff documented whether a patient had received services within six months of their index ED visit. Advocates then aggregated the linked dataset by hospital, gender, race/ethnicity, and whether or not the patient had been given the intervention in the ED; due to agency policy, the resulting data could not be linked at the individual level to hospital data. As a result, all analyses including advocacy data were conducted separately and did not include the additional variables in the main hospital dataset.

Outcome Variables

This study examines two key outcome variables: (1) DV-related referrals given during the ED visit; and (2) subsequent patient contact with advocacy services. DV-related referrals indicate that the provider gave the patient tangible information or resources related to DV, such as an educational pamphlet, a phone number for an advocate, or a voucher for emergency shelter. Medical follow-up visits and police referrals were not considered DV-related referrals, although referrals to restraining order clinics or other legal services were included. Contact with advocacy services indicated that the advocacy agency staff documented contact with an individual of the same first and last name within six months of the index ED visit. Contact included in-person advocacy services from on-site advocates or 27 on-site partner agencies as well as phone-based advocacy services from agency advocates.

Data Analysis

<u>Main effects</u>

We grouped ED visits into three categories: visits that occurred (1) prior to DVRR implementation at the hospital; (2) after DVRR implementation but at which time the patient was provided standard care instead of the intervention; and (3) after DVRR implementation and at which time the intervention was administered. We chose individual visits as the unit of analysis because patients could have been victimized by multiple perpetrators or situations during the course of the study, requiring separate interventions from ED and advocacy personnel. We used general linear models stratified by hospital to calculate estimates and 95% confidence intervals for changes in patients' relative likelihood of receiving DV-related referrals and advocacy services before and after the intervention was implemented. We repeated analyses among visits that occurred after the intervention was implemented, stratified by whether or not the provider administered it

during the visit. To adjust for hospital-specific effects of the intervention, all models included indicator variables for each hospital and interaction terms between the hospital and implementation status. Multiple baseline graphs visually demonstrate any change in patient referral and advocacy outcomes after the intervention was implemented at each hospital.

External validity

The multiple baseline graphs also convey a measure of the main effects' external validity. They enable examination of changes in referrals and advocacy visits at Hospital 1 during the time period when Hospitals 2 and 3 were not implementing (i.e, their baselines), and vice versa.

First, we used logistic regression and traditional chi-square tests to assess all three hospitals for changes in DV referrals and advocacy services both during their own rollout and during the other hospitals' implementation of the intervention. Hospitals 2 and 3 implemented DVRR almost simultaneously, so they were considered to have the same implementation period for this test. Any significant results from this test suggest the presence of a confounding external factor affecting the measures of all study hospitals during an individual hospital's rollout.

Second, we used the associated Mantel-Haenszel chi-square test statistic to compare whether changes observed in Hospital 1 were significantly different from Hospitals 2 and 3 at each implementation point[33]. This modification to a traditional chi-square test statistic gives weight to clusters (i.e., the three study hospitals) based on the number of data points each contains. This was used due to disparate sample sizes between the three hospitals. When cell sizes of 0 precluded Mantel-Haenszel estimation, we used a Breslow-Day test of homogeneity between the two remaining hospitals with a Tarone adjustment to asymptotically limit the resulting distribution.[34,35] We set a 95% confidence interval to assess significance; any significant findings suggest that the intervention caused the change in measured outcomes.

Internal validity

We also used Mantel-Haenszel chi-square tests to assess the internal validity of the main effects of this study. We regressed referrals to non-DV resources such as mental health care, general psychosocial care, and all non-police legal support against the two main predictors in this study: whether the hospital had implemented DVRR and whether it had been offered to the patient during their visit. Any significant results from these tests suggest something akin to a placebo effect, wherein heightened training, awareness, or other aspects of ED care related to the intervention influenced the rate of referrals within the ED without specificity to DV.

We compared these results to the intervention's association with DV-related referrals using one-way MANOVA. Any significant results from these tests suggest that the intervention was associated with a significantly greater change in DV-related referrals than non-DV-related referrals. These results speak to the specificity of the intervention in addressing DV. We used Stata statistical analysis software (version 14.2) for all analyses.[36]

Results

Sample Characteristics

Patient characteristics varied greatly across this sample (Table 1). More patients screened positive at Hospital 1 and Hospital 2 than at Hospital 3. Before and after implementation, significant differences persisted between hospitals in rates of DV-related

referrals, connection to advocacy services, and rates of sexual assault among DV patients. However, among the subset of patients who received the DVRR intervention, these differences disappeared. Implementing the intervention was also associated with a significantly lower rate of DV-related visits at both Hospitals 2 and 3. Providers at Hospital 2 administered the intervention to a significantly lower proportion of DV-affected patients than Hospital 1 or Hospital 3.

Table 1: Description of DV referrals given and advocacy services received among patients with confirmed DV cases before/after hospitals implemented DVRR								
**Before DVRR implementation**								
	Hospital 1 n=333		*Hospital 2 n=224*		*Hospital 3 n=73*			*Total n=630*
	%	*n*	*%*	*n*	*%*	*n*	*%*	*n*
*DV referrals***	72.97%	243	2.68%	6	9.59%	7	40.63%	256
*DV services***	18.34%	62	3.59%	8	5.97%	4	11.74%	74
Repeat visits	25.23%	84	35.87%	80	---	---	26.03%	164
*Sexual assault***	13.51%	45	2.23%	5	7.04%	5	8.73%	55
**After DVRR implementation**								
**Total**								
	Hospital 1 n=1474		*Hospital 2 n=161**		*Hospital 3 n=29**			*Total n=1664*
	%	*n*	*%*	*n*	*%*	*n*	*%*	*n*
*DV referrals***	76.87%	1133	18.01%	29	41.38%	12	70.55%	1174
*DV services**	24.63%	363	13.04%	21	10.34%	3	23.26%	387
Repeat visits	25.64%	378	21.74%	35	---	---	24.82%	413
*Sexual assault***	15.20%	224	4.35%	7	24.14%	7	14.30%	238
DVRR not administered								
	Hospital 1 n=678		*Hospital 2 n=139*		*Hospital 3 n=17*			*Total n=833*
*DV referrals***	57.96%	393	8.63%	12	17.65%	3	26.40%	408
DV services	11.65%	79	7.19%	10	0.00%	0	2.82%	89
Repeat visits	27.43%	186	23.19%	32	---	---	26.17%	218
*Sexual assault***	17.85%	121	4.32%	6	21.05%	4	15.73%	131

DVRR administered								
	Hospital 1 n=796		*Hospital 2 n=22*		*Hospital 3 n=12*			*Total** n=829*
DV referrals	92.96%	740	77.27%	17	75.00%	9	92.64%	768
DV services	35.68%	284	50.00%	11	25.00%	3	35.90%	298
Repeat visits	24.12%	192	13.64%	3	---	---	23.52%	195
Sexual assault	12.94%	103	4.55%	1	25.00%	3	12.91%	107
*Note: *p<0.05; **p<0.01. Repeat visits not measured at Hospital 3.*								

Main Results

DVRR implementation was associated with a significantly higher likelihood of the patient receiving a referral to DV services at Hospitals 2 and 3 (Table 2). Intervention implementation was associated with a significantly greater likelihood of receiving advocacy services at all three study hospitals. After implementation, patients who received DVRR during their ED visit had a significantly greater likelihood of receiving referrals at all study hospitals. At Hospitals 1 and 2, patients who received the intervention also had an increased likelihood of receiving advocacy services. At Hospital 3, after DVRR had been implemented, only patients who received it ultimately made contact with advocacy services, thus the likelihood ratio could not be estimated for this site.

Table 2. Relative proportion of patients receiving DV referrals and advocacy services by DVRR status						
**Before vs after DVRR implementation (total)**						
	Hospital 1 n=333		*Hospital 2 n=224*		*Hospital 3 n=73*	
	DV referrals	DV services	DV referrals	DV services	DV referrals	DV services
Proportion before DVRR (a)	0.73	0.18	0.03	0.04^	0.09	0.06
Proportion after DVRR (b)	0.77	0.25	0.19	0.13	0.41	0.1
Relative proportion (b/a) (95% CI)	1.05 (0.98-1.13)	1.34* (1.05-1.71)	6.72** (2.86-15.82)	3.55** (1.61-7.81)	4.47** (1.96-10.19)	1.68** (0.40-7.03)
**After DVRR implementation: DVRR administered vs not administered**						
	Hospital 1 n=678		*Hospital 2 n=139*		*Hospital 3 n=17*	
	DV referrals	DV services	DV referrals	DV services	DV referrals	DV services

Proportion given DVRR (a)	0.58	0.14	0.02	0.07	0.08	0
Proportion not given DVRR (b)	0.93	0.36	1.00	0.44	1.00	0.25
Relative proportion (b/a) (95% CI)	1.60** (1.50-1.71)	2.55** (2.13-3.05)	8.95** (4.98-16.09)	6.16** (2.93-12.95)	4.64** (1.60-13.43)	---
*Notes: *p<0.05; **p<0.01. ^Patients had a higher measured relative likelihood of reaching advocacy services (0.04) than being referred to those services (0.03) prior to DVRR implementation. This was not tested for significance.*						

Study hospitals had vastly different rates of DV referral and subsequent receipt of advocacy services prior to implementing the intervention (Figure 2). The intervention was significantly associated with increased rates of referral at Hospitals 2 and 3 and increased connection to DV advocacy at all three study hospitals.

Figure 2: Multiple baseline depiction of DV referrals and advocacy services before/after DVRR implementation in EDs

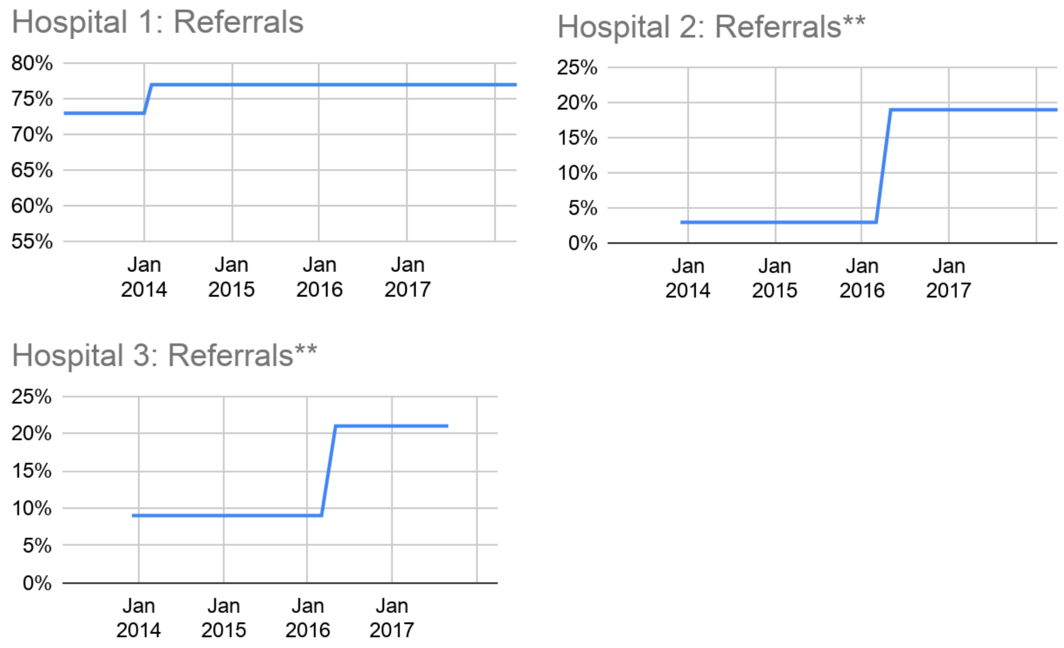

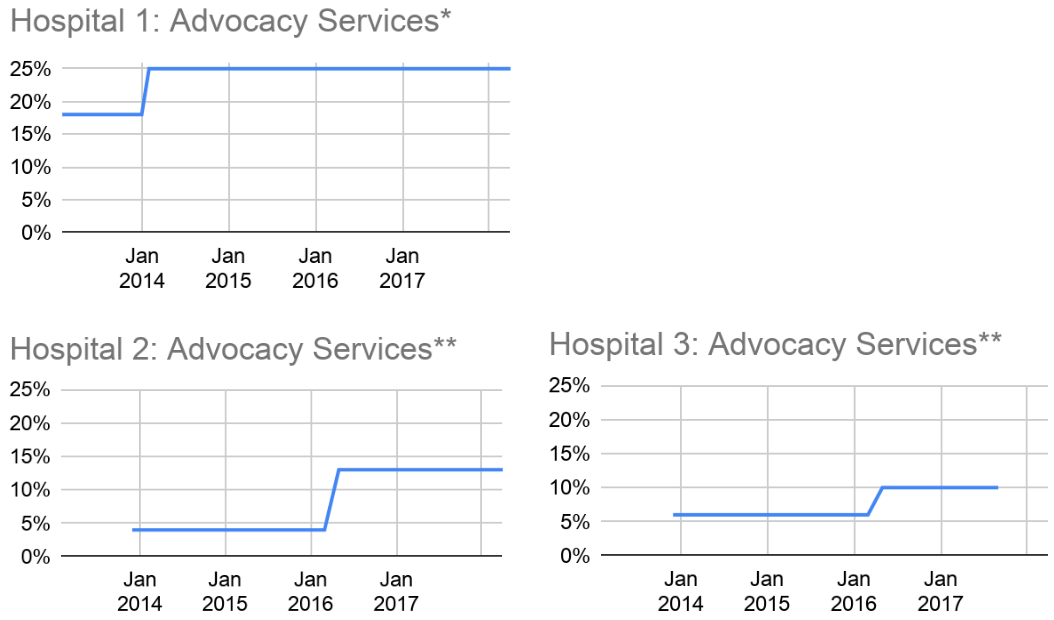

*p<0.05; **p<0.01

These main effects were combined with measures of internal and external validity to assess any causal link between the DVRR intervention and a targeted increase in DV referrals at each hospital. The multiple baseline diagrams in Figure 2 further suggest a preliminary indication of external validity. In these figures, while Hospitals 2 and 3 experienced a significant shift in DV referrals and advocacy services after implementing the intervention in April and May 2016, Hospital 1 appears to have experienced no change at this time. This is also true of the significant change in advocacy services received by Hospital 1 patients after implementing the intervention in February 2014; Hospitals 2 and 3 do not show a similar change at this time point.

In Table 3, these differences are quantified using logistic regression models and Mantel-Haenszel chi-square tests. These tests found that the change in DV referrals and advocacy services differed significantly between Hospital 1 and Hospitals 2 and 3 when Hospitals 2 and 3 implemented the intervention. Because of sample size limitations, Hospital 3 was excluded from analyses during the Hospital 1 implementation period, and, as stated earlier, a Breslow-Day test for homogeneity with a Tarone adjustment was used to examine any change in DV referrals at Hospital 2 after Hospital 1 implemented DVRR. This test found that DV referral rates did not significantly differ between Hospitals 1 and 2 at the time of Hospital 1 implementation. This is consistent with the main finding that Hospital 1 did not experience a significant change in referral rates after implementing DVRR.

Table 3. Relative proportion of patients receiving DV referrals and advocacy services by DVRR status
**Before vs. after Hospital 1 implementation**

	DV referrals			*DV services*		
	Hospital 1	Hospital 2	Hospital 3	Hospital 1	Hospital 2	Hospital 3
Odds ratio	1.05	0.78	---	Could not be calculated due to sample size limitations		
95% CI	(0.98-1.13)	(0.10-36.09)				
Mantel-Haenzel chi-square statistic	1.10 (Breslow-Day)		---			
**Before vs. after Hospital 2 and 3 implementation**						
	DV referrals			*DV services*		
	Hospital 1	Hospital 2	Hospital 3	Hospital 1	Hospital 2	Hospital 3
Odds ratio	1.03	6.67**	11.00**	1.19	3.92**	1.75
95% CI	0.80-1.32	2.86-17.29	3.20-40.21	0.91-1.56	1.61-10.48	0.24-11.05

Taken together, these findings suggest that no extraneous factors other than DVVR implementation affected DV referrals or advocacy services received during the implementation of DVRR at study hospitals.

Finally, we calculated the odds ratios associated with referrals to both DV and non-DV resources before and after each hospital implemented the intervention (Table 4). After implementation, we stratified analyses by whether or not the intervention was offered in individual visits. This model comprised all three hospitals and suggested that the impact of implementing the DVRR intervention was significant for both DV-related referrals and non-DV related referrals ($p<0.01$). This finding was driven by the lack of a significant effect among the large sample at Hospital 1, at which implementation was associated with no increase in the odds of any referrals, and by the significant increase in both DV and non-DV referrals at Hospital 2.

Table 4: Difference in odds between DV referrals and non-DV referrals by DVRR status				
**Before vs. after DVRR implementation (total)**				
	Hospital 1	*Hospital 2*	*Hospital 3*	*Mantel-Haenszel combined*

	DV referrals	Non-DV referrals	DV referrals	Non-DV referrals	DV referrals	Non-DV referrals	DV referrals	Non-DV referrals
Odds ratio	1.23	1.04	7.98**	0.76	7.07**	0.64	1.65**	0.99
95% CI	0.93-1.62	0.79-1.38	3.13-24.02	0.38-1.49	2.12-24.37	0.01-6.87	1.30-2.10	0.77-1.26
F statistic (MANOVA)	1.25		14.71**		9.75**		96.73** (unweighted)	
**After DVRR implementation: Administered DVRR vs not administered DVRR**								
	Hospital 1		*Hospital 2*		*Hospital 3*		*Mantel-Haenszel combined*	
	DV referrals	Non-DV referrals	DV referrals	Non-DV referrals	DV referrals	Non-DV referrals	DV referrals	Non-DV referrals
Odds ratio (95% CI)	9.55**	0.60**	35.98**	2.15	21.00**	0	10.34**	0.62**
95% CI	6.94-13.27	0.48-0.76	10.07-141.39	0.46-8.00	2.26-258.10	0.00-0.00	9.41-17.19	0.50-0.79
F statistic (MANOVA)	157.48**		47.95**		12.15**		249.26**	
Notes: **p<0.05; **p<0.01*								

After implementation, odds of receiving DV referrals were significantly higher among patients who received the DVRR intervention; this finding was consistent in both the Mantel-Haenszel combined model and at all three individual hospitals (p<0.01). The odds of DV-affected patients receiving referrals to non-DV services significantly decreased in both the Mantel-Haenszel combined model (p<0.01) and at Hospital 1. Individual and combined MANOVA results suggest that the DVRR intervention was associated with a significantly greater impact on DV than non DV-referrals (p<0.01). These results suggest that no other factor internal to the hospitals or study measurements led to a general increase in all referrals. Instead, the intervention was associated with a targeted increase in DV referrals at each hospital. This lends support to the internal validity of the main effects of this study.

Limitations

The small sample size at Hospital 3 and the shorter baselines at Hospitals 2 and 3 posed the most significant limitations to this study. Because of the small sample size at Hospital 3, the estimated results for this hospital have wide confidence intervals. Due to this and the shorter baselines at Hospitals 2 and 3, we were limited in the tests we could conduct for external validity during Hospital 1 implementation.

Internal validity could be assessed for DV referrals but not receipt of advocacy services. Doing so would have required outcome data from non-DV agencies, such as mental health services or substance abuse services, that were unavailable for this study. Because results indicated the DVRR intervention was negatively associated with referrals to non-DV resources, future research measuring services received from other referral destinations would provide further insight into the main effects' internal validity.

Data linkage limitations also affected this study. Because first and last names provided the basis for data linkage between hospitals and the advocacy agency, any differences in spelling between ED and advocacy records would have resulted in a mismatch. Similarly, any advocacy clients with the same first and last name as a hospital patient seen within six months of the hospital visit would have been falsely recorded as a match; no study measures assessed the magnitude of this limitation.

This study only evaluated advocacy contact at the agency that received all referrals from the intervention. It did not evaluate the nature or length of advocacy services received, nor advocacy contact at any other advocacy agencies. This agency had served as the primary referral destination for Hospital 1 and a major referral destination for Hospitals 2 and 3 before the intervention, but it is possible that findings may reflect some rerouting of patients who would have made contact with other DV agencies. However, Hospitals 2 and 3 provided DV referrals to a very low proportion of patients without the DVRR intervention. As a result, it is unlikely many patients would have sought services from an alternative DV agency.

Discussion

The main findings of this study suggest the DVRR intervention is associated with significantly higher rates of patient connection to DV advocacy services through more effective referrals. The tests for external validity suggest that no other global or external factors were responsible for this increase, and the tests for internal validity suggest that this increase also cannot be explained by other factors associated with implementing the intervention, such as increased provider training and awareness.

DVRR is a digital warm handoff intervention for DV, grounded in evidence-based practice. We found that implementing this intervention was associated with an increase in the rate of both referrals and advocacy services offered to DV-affected patients. Upon implementing the intervention, two of the three study hospitals experienced a significant increase in DV referrals, and all three experienced a significant increase in advocacy services received. After implementation, providers administered the DVRR intervention to eligible patients at their discretion. Thus, we also measured differences by whether patients received the intervention. Patients who received the DVRR intervention were one and a half to nine times as likely to receive DV referrals and advocacy services. Measures of internal and external validity suggest a likely causal relationship between the DVRR intervention and increased rates of DV referrals and receipt of advocacy services.

These findings support the utility of eHealth warm handoffs to connect DV-affected patients to advocacy services. In each ED, three to six times as many patients connected with advocacy after the hospital integrated a digital warm handoff into their DV care. This is consistent with previously described research, findings from which suggest that direct connections to supportive services and advocacy improved patient connections, outcomes and financial feasibility.[6,21,37] The digital delivery of the present intervention integrates a

warm handoff into the existing environments and workflows of both hospital and advocacy agency staff.

Baseline data suggest that the standard of care at Hospitals 2 and 3 often left patients without support. Despite near-universal screening at Hospital 2, fewer than 3% of patients who disclosed DV received any referral to services. As 4% of Hospital 2 patients reached advocacy, many did so despite receiving no referrals or resources from the hospital. Before implementing the intervention, each of the three study hospitals used different DV referral protocols, as well. This resulted in significantly different referral rates. Of known DV-affected patients, for example, Hospitals 1, 2, and 3 had referral rates of 72%, 3%, and 10%. As stated earlier, these inconsistencies are emblematic of the ambiguous, inconsistent guidance around DV intervention across healthcare systems described in a systematic review of 35 studies examining healthcare-based DV interventions.[10] As a result, it is unsurprising that few patients connected with advocacy services before the intervention (18%, 4% and 6% at Hospitals 1, 2 and 3, respectively). After implementing the intervention, DV-affected patients were significantly more likely to receive DV-related referrals at Hospitals 2 and 3 and advocacy services after referral from all three hospitals. This may be, in part, due to ways DVRR design differs from usual care. This intervention offers step-by-step guidance to providers and patients and transfers the burdens of seeking and administering DV care from patients and providers to professional DV advocates.

Recent studies suggest that eHealth interventions are not a silver bullet to prevent future harm to DV-affected patients.[13,24,38,39] Rather, eHealth interventions tend to add value when they facilitate intervention that otherwise would have required prohibitive financial, time, personnel, or other resources.[26] In contrast to typical eHealth interventions, which digitize interventions with minimal impact on patient wellbeing, DVRR streamlines delivery of a best practice intervention using eHealth.[21,37] By doing so within existing EDs and DV advocacy infrastructure, it enables a warm handoff between providers and DV advocates that otherwise may not have occurred.

Yet even best-practice interventions leave many DV-affected ED patients unserved. One reason for this is a lack of DV detection in EDs. Inconsistent screening practices within and among EDs render DV identification and intervention highly variable.[10,26] Even when providers detect DV and administer direct-to-advocacy interventions, patient prospects of advocacy care are bleak. The two studies to measure this outcome suggest that 27% and 65% of patients (in sample sizes of 41 and 122) never made contact with advocacy services.[6,38] In the present study, about 64% of the 829 patients who received the intervention never reached advocacy services, in addition to 97% of the 833 patients who did not receive the intervention. Further, providers administered the intervention to only about half of DV-affected patients at Hospitals 1 and 3, and only 15% of DV-affected patients at Hospital 2. These low rates highlight the vast number of DV-affected patients who received no DV-focused care from ED providers or advocates, in spite of the elevated mortality risks they face. As noted previously, the average lethality risks of patients given DVRR in one of the study hospitals noted that 61% of patients experienced a high (28%) or extremely high (33%) risk of intimate partner homicide.[8,9]

Future research may focus on unanticipated consequences of enhanced DV interventions such as digital warm handoffs. For example, the introduction of the DVRR intervention to Hospitals 2 and 3 was associated with a significant decrease in the rate of patients whose medical records indicated DV; it is unlikely this decrease reflects an

actual decrease in DV-related visits to these hospitals. In addition, patients who received the present intervention were less likely to receive other mental health or legal aid referrals. Providers may have anticipated advocates would provide these services through their work with the patients. Yet, many of these patients did not receive follow-up advocacy services and thus received no resources or referrals at all. This scenario would particularly restrict care for DV-affected patients who wanted to be connected to advocacy care but declined DVRR, whether due to the incorporated mandated police report or other reasons. Future research should examine these unintended consequences of care, as well as the trajectories of DV-affected patients after they seek ED care, including barriers to acting on referrals, the nature and impact of advocacy care, future safety, and rates of attempted or completed intimate partner homicide.

Taken together, the findings from this study suggest that digital warm-handoff interventions such as DVRR may causally improve DV-affected ED patients' connection to advocacy services. This highlights a promising, much-needed advance in the comprehensive care of a uniquely vulnerable, high-risk population often seen in the ED.

Acknowledgments:
The authors would like to thank Hospital 1 and Hospital 3 partners for facilitating hospital IRB efforts, Hospital 1 and Hospital 2 partners for providing on-site working space and access to medical records, Hospital 3 and agency partners for abstracting records, and Hillary Larkin for providing access to the DVRR database. Additionally, we acknowledge Cheung Hang-Fok, Adeline Nguyen, Michiko Saka-Scott, Eugenia Rodriguez and Cecilia Villalobos for their assistance with data collection; Emily Ozer for reviewing the initial research design and reviewing the final version of this manuscript; and Gilead Sciences, Inc for providing funding for this research (Grant #00495).
