# Supplementary material for: Access to domestic violence advocacy by race, ethnicity and gender: The impact of a digital warm handoff from the emergency department
Source: PLoS One. 2022 Mar 18;17(3):e0264814. doi: 10.1371/journal.pone.0264814 (PMC8932576; doi:10.1371/journal.pone.0264814)
Supplement: Attachment — Submitted filename: Reviewer Responses - round 2.docx [file pone.0264814.s005.docx]

*The authors would like to thank the reviewers/editor for their thoughtful, constructive comments. Their suggestions have been implemented as described in green italic text below.*

Reviewer #1: This is an important study of ED patients with DV, their receipt of a digital warm handoff intervention called DVRR, and their receipt of advocacy services. I have several questions and comments to improve clarity.

Abstract:
1. Background: I suggest changing first sentence to “racial/ethnic minority survivors of domestic violence (DV) referred from emergency departments (Eds) face barriers connecting with advocacy services due to systemic inequalities.” Their inequities in healthcare access may not be the same as those found in advocacy services. I also suggest describing DVRR as an electronic reporting system to fulfill mandatory reporting requirements to law enforcement, with report also going to DV advocacy agency.

*The proposed changes have been implemented.*

2. Methods: Describe time frame (2014-2018) for study. Briefly describe how DV-associated ED visits were defined. I recommend not describing sample sizes (1366, 740, 323) in methods section as those are results. You could state something like “We assessed 2014-2018 ED visit chief concern for DV, measured if DVRR occurred, and if patient names matched DV advocacy agency records of survivors using services”. I would suggest giving sample size for number used in logistic regression analyses and number used in predicted probability analyses.

*Each of these proposed changes has been implemented.*

Manuscript text
Introduction
1. 2nd paragraph: You write that “standard of care for DV often does not extend beyond hospital screening; when it does, it typically consists of printed educational material or a phone number to a community-based advocacy agency…”, and I think you are meaning that standard of care for DV includes identification of DV but does not necessarily include providing support and resources, or follow-up to see if patient used advocacy agency services. I would clarify this.

*The paragraph has been clarified per this exceptionally helpful comment.*

2. 2nd paragraph: You write “…warm handoff, in which the ED provider personally transfers the survivor’s DV care to a DV advocate.” A transfer of care in medical care more typically means physical movement of the patient location. A warm handoff might be better described as a healthcare provider describing DV advocacy agency services and possibly calling the DV advocacy agency while in the patient room. This may include a DV advocacy agency staff member coming to the patient location for an in-person provision of support and resources. I would clarify this.

*The paragraph has been revised to clarify this information.*

3. Race and ethnicity, and Gender sections: I recommend condensing this into 1 paragraph. The focus of the paper is ED visits, DVRR and DV advocacy visit. The first 2 paragraphs describe ED visits and DV advocacy, and the last paragraph describes DVRR.

*These two sections have been condensed into a single section with three paragraphs. The key findings in this paper highlight differential results by race, ethnicity and gender and this section has been condensed to more concisely presage those findings.*

Results
1. I recommend changing “Analysis” header (between first and second paragraphs) to “DVRR and receiving advocacy services by race/ethnicity and gender”. Otherwise, the reader will think this is the methods section analysis plan

*The heading has been revised per this section.*

2. Last paragraph: what does likelihood difference assist reader in knowing?

*This paragraph has been revised to read “Female survivors were significantly more likely than male survivors to subsequently connect with advocacy services (Fig 3). DVRR use appears to narrow that gap within this sample.”*

Table 2:
1. I would put 95%CI with odds ratio in 1 row.

*This table has been reformatted and the preceding paragraph has been updated accordingly*

2. Why is the reference group baseline odds ratio (white, female) 0.12? Shouldn’t it be 1.0 as it’s the reference group?

*This information has been removed from the table and inserted in the preceding paragraph*

Figures 1 and 2: what does statistical test for % increase add? I think you can remove it. It’s otherwise too much information. It’s obvious from the table that there is a visually large increase by DVRR.

*These tests have been removed.*

S1 Appendix: can you explain more why this appendix is needed? Doesn’t Figure 2 adequately show differences in DVRR status by race and ethnicity?

*This appendix has been removed.*

Discussion: Paragraph 3: the authors describe how 5-question Danger Assessment “is an important step toward improving the strength of the Danger Assessment component of DVRR in reducing bias and providing culturally appropriate care.” I think the using 5-question DA will help provider time in completing the DA, and therefore completing the full DVRR assessment. It may increase DVRR administration from 54% to something higher.

*This consideration has been added.*

Strengths and limitations: Paragraph 3: can you describe the “data restrictions that made it impossible to study additional groups that…experience heightened barriers to DV response or services”. Can you explain more about those data restrictions?

*The sentence described has been revised and expanded to state the following:*

*To protect client privacy at the advocacy agency, covariate data collection was restricted a priori to variable combinations with cell sizes of 2 or greater. As a result, researchers were limited to six major covariate indicators (DVRR administration, male or female gender, Black, Latinx or non-Latinx white race/ethnicity). This required excluding certain populations with small sample sizes (e.g., Asian, Pacific Islander, Indigenous), and not specifying other relevant characteristics either due to a small sample size (e.g., LGBTQ+ identity) or non-categorical data structure (e.g., age).*

Reviewer #2: The revision addresses my original comments, and I have no further comments.
